# Anti-Inflammatory, Antioxidant, and Genoprotective Effects of Callus Cultures Obtained from the Pulp of *Malus pumila* cv Miller (Annurca Campana Apple)

**DOI:** 10.3390/foods13132036

**Published:** 2024-06-27

**Authors:** Federica Gubitosa, Daniele Fraternale, Leila Benayada, Roberta De Bellis, Andrea Gorassini, Roberta Saltarelli, Sabrina Donati Zeppa, Lucia Potenza

**Affiliations:** 1 Department of Biomolecular Sciences, University of Urbino Carlo Bo, 61029 Urbino, Italy; f.gubitosa@campus.uniurb.it (F.G.); daniele.fraternale@uniurb.it (D.F.); l.benayada@campus.uniurb.it (L.B.); roberta.debellis@uniurb.it (R.D.B.); roberta.saltarelli@uniurb.it (R.S.); sabrina.zeppa@uniurb.it (S.D.Z.); 2 Department of Humanities and Cultural Heritage, University of Udine, 33100 Udine, Italy; andrea.gorassini@uniud.it

**Keywords:** Annurca apple, callus production, secondary metabolites, triterpenic acids, biological activities, functional foods

## Abstract

Apples are rich in phytochemicals useful for human health. However, environmental factors can greatly affect the accumulation of these compounds. To face this problem, the callus culture technique was used to obtain large quantities of phytochemicals. Specifically, two callus cultures were obtained from ripe Annurca apple pulp (*Malus pumila* cv Miller) and cultivated under different light conditions: darkness and an 18-h photoperiod. The hydro-alcoholic extracts from the calli underwent analysis using GC-MS, GC-FID, and HPLC-DAD-ESI-MS^n^ to determine the qualitative and quantitative content of phenolic and triterpenic acids. The study revealed the predominant presence of triterpenic compounds in both calli. Furthermore, we investigated their radical scavenging and antioxidant activities through DPPH, ABTS, ORAC assays, and lipoxygenase inhibition activity. Genoprotection was evaluated via nicking assay, and the anti-inflammatory effect was investigated via Griess assay on LPS-injured murine macrophages. All the analyses performed were compared with peel and pulp hydroalcoholic extracts. The results showed that both calli primarily show anti-inflammatory activity and moderate antioxidant effect and can protect DNA against oxidative stimuli. This data encouraged further research aimed at utilizing callus as a bioreactor to produce secondary metabolites for use in preventive and therapeutic applications to combat acute or chronic age-associated diseases.

## 1. Introduction

The concept that consuming fruits and vegetables improves health has been well-established for several years. Apples have been the subject of numerous health studies worldwide due to their phytochemical content [1]. Literature data report that several chronic diseases (e.g., cardiovascular and obstructive pulmonary disease) can be prevented by consuming apples and their components [2].

Annurca apples have attracted significant attention among the various apple varieties. Its historical significance traces back to the ancient Roman era, rendering it a preeminent cultivar in southern Italy. It is important to note that this variety is the most extensively cultivated in the Campania region. Given the significant economic value of the Annurca cultivar to the Campania region, the ‘Istituto Sperimentale di Frutticultura’ in Ciampino (Rome) established a research program in 1970 to address the cultivar’s inherent limitations. This endeavor encompassed comprehensive studies of its biological characteristics, breeding techniques, and optimal storage conditions. Furthermore, a formal proposal was submitted to the European Council for the conferral of ‘Protected Geographical Indication’ (PGI) status under European regulation number 2081/92. This recognition seeks to facilitate the marketing of the Annurca apple cultivar at both the local and international levels [3,4,5]. The Annurca apple is distinguished by its unique ripening process. Following harvest, the fruit undergoes a postharvest reddening treatment using traditional structures known as ‘melai’. These structures are composed of a layer of straw placed on the ground to provide support for the fruit. Subsequently, shading nets are employed to mitigate solar radiation and regulate temperature. During October, the apples are harvested and subsequently positioned on a bed of straw on the ground, which is then consistently moistened. Upon the fruits’ surface turning red, they are manually rotated to ensure an even reddening. Depending on weather conditions and fruit characteristics, this treatment can range from 20 to 30 days. Following the reddening process, this cultivar demonstrates excellent storage properties, retaining firmness and emanating a pleasant aroma [3,5]. Several studies have shown that traditional reddening treatment may increase bioactive compounds like flavonoids and anthocyanins in Annurca fruits during storage, leading to significant antioxidant activity [5].

Thus, apples contain a high concentration of phytochemicals, which may vary depending on various factors, including apple cultivar, harvest, storage, and processing [6]. Plant tissue culture represents an enticing alternative to produce vital pharmaceutical secondary metabolites. This technology offers numerous advantages over traditional agricultural methods. These advantages include consistent crop quality and yield, the capacity to fulfill the demand for specific plant compounds, production under rigorously controlled conditions, and the avoidance of contamination with pesticides, herbicides, agrochemicals, or fertilizers [7]. Among the various *in vitro* propagation techniques, the most widely used is based on forming callus cultures from adult plant cells. A callus represents an aggregation of irregular, undifferentiated cells capable of generating metabolites akin to those present in the parent plant. Moreover, these callus cells exhibit totipotency and plasticity characteristics, thereby enabling them to modulate their metabolic processes and adapt their growth patterns in response to varying environmental conditions [8]. These callus properties can be used to enhance the levels of beneficial secondary metabolites in fruits and vegetables, which can contribute to human health [9]. To use callus cultures as bio-sources of secondary metabolites to be proposed as nutraceuticals, functional foods, or functional ingredients, the present study aimed to set up callus production from the ripe pulp of *Malus pumila* cv Miller (Mela Annurca Campana) and perform the first chemical characterization and investigation of the biological properties of the callus-derived hydroalcoholic extracts.

## 2. Materials and Methods

### 2.1. Chemical and Reagents

Extraction and derivatization solvents were of analytical grade and were obtained from Sigma-Aldrich (Milan, Italy). Cholesterol (GC-FID Internal Standard; IS), β-sitosterol, ursolic, and oleanolic acids, used as standards, and Sylon BFT [Bis-(trimethylsilyl)-trifluoroacetamide (BSTFA) with 1% trimethylchlorosilane (TMCS)], employed as a silylating reagent, 6-benzylaminopurine (BA), 1-naphthaleneacetic acid (NAA), and agar were also obtained from Sigma-Aldrich (Milan, Italy). Methanol (MeOH) and formic acid (HCOOH) for HPLC-MS, (+)-catechin, (−)-epicatechin, 5-caffeoylquinic acid (chlorogenic acid), and 3-hydroxycinnamic acid (HPLC-DAD Internal Standard; IS) were purchased from Sigma-Aldrich (Milan, Italy). Quercetin-3-*O*-galactoside and procyanidin B2 were obtained from ExtraSynthese (Lyon, France). Quercetin-3-*O*-arabinoside, quercetin-3-*O*-xyloside, and quercetin-3-*O*-rhamnoside were purchased from Carbosynth (Berkshire, UK). Milli-Q-grade water was produced by the Elgastat UHQ-PS system (ELGA, High Wycombe Bucks, UK). Solid-phase extraction (SPE) columns ISOLUTE C18, 1 g, 6 mL were from Biotage (Milan, Italy). Reagents for antioxidant activity experiments and nicking assays were purchased from Sigma-Aldrich (Milan, Italy).

### 2.2. Plant Material and Callus Cultures

Mature apples were purchased from large retailers in December 2019. The fruits come from the production consortium ‘Mela Annurca Campana’ IGP. Ripe Annurca apple pulp was used to start callus culture induction. The culture was carried out in the laboratories of the Botanic Garden at the University of Urbino Carlo Bo. Before proceeding with the pulp explants, the fruits underwent surface sterilization by ethanol washing and burning off under a sterile laminar flow hood. The apples were subsequently sliced using a sterile scalpel, and discs 5 mm in diameter and approximately 3 mm in height at 0.5 cm from the fruit skin were taken as explants using a sterilized corkscrew [10]. The explant cultures were conducted using Murashige and Skoog culture medium plus 30 g/L sucrose and supplemented with 6-benzylaminopurine (BA, Sigma-Aldrich) (8.8 µM) plus naphthaleneacetic acid (NAA, Sigma-Aldrich) (10.0 µM). The culture medium was brought to pH 5.8 before the addition of agar into Petri dishes in the amount of 30 mL per Petri dish. Cultures were kept in the dark at 25 ± 2 °C. We named this culture ‘dark callus’ (DC). Subcultures of callus were taken every 28 days from the same culture medium. This optimal culture condition for obtaining callus from ripe Annurca apple pulp was maintained to obtain material by subculturing every 28 days in fresh culture medium each time. To assess any changes in the phytocomplex composition, the calli were also maintained in an 18-h photoperiod obtained with Cool White fluorescent light at 19 µmol/m^2^ s, at a constant temperature of 25 ± 2 °C. We named this last culture ‘light callus’ (LC). The material collected from the sub-cultures was kept in a freezer at −20 °C until it was freeze-dried and then extracted, and the extracts were used for chemical analysis and various biological tests. To conduct a comparative analysis of naturally occurring metabolites in the pulp and peel of apples, several other fruits were also selected for inclusion in the study. Subsequently, the apple peel and pulp under consideration were promptly subjected to freeze-drying and then stored in hermetically sealed containers at a temperature of −20 °C until required.

### 2.3. Preparation of Extracts from Peel, Pulp, and Calli of Annurca Apple

Each 200 mg lyophilized sample underwent suspension in 11.5 mL of 70% ethanol and homogenization using a Potter-type homogenizer. The resulting homogenate was transferred into 50 mL tubes and subjected to overnight stirring at +4 °C. Subsequently, each tube underwent centrifugation at +4 °C and 13,000 rpm for 45 min. The resultant supernatant was then centrifuged at +4 °C and 4000 rpm for 30 min and collected in 50 mL empty tubes. The pellet was resuspended in 11.5 mL of 70% ethanol, stirred for one hour, and then centrifuged at +4 °C and 13,000 rpm for 45 min. The supernatant from this centrifugation was again collected and further centrifuged at +4 °C and 4000 rpm for 30 min. The resulting supernatants were concentrated and subjected to lyophilization using a Speed Vac Concentrator (Thermo Fisher Scientific, Waltham, MA, USA). The lyophilized samples were stored at −20 °C and reconstituted in 70% ethanol before use.

### 2.4. Chemical Characterization of Extracts from Peel Pulp and Calli of Annurca Apple

Each lyophilized sample (30 mg) and 30 μL of the IS solution (3-hydroxycinnamic acid, 100 μg/mL in MeOH) was dissolved in H_2_O with 0.4% formic acid (2 mL) and loaded onto a C18 SPE column previously conditioned with 10 mL of MeOH with 0.4% formic acid and 10 mL of H_2_O with 0.4% formic acid. After loading, the column was washed with 10 mL of H_2_O with 0.4% formic acid, and the phenolic fraction was eluted with 5 mL of MeOH with 0.4% formic acid. The solvents were removed, and the residue was diluted in 1 mL of H_2_O/MeOH 9:1 (*v*/*v*) with 0.4% formic acid and transferred to an autosampler bottle for HPLC-DAD-ESI-MS^n^ analysis. Chromatographic analysis was performed with a Dionex Ultimate 3000 UPLC (Thermo Fisher Scientific, MA, USA) equipped with a thermostatic autosampler and a column oven. Chromatographic separation was performed with an InfinityLab Poroshell 120 EC-C18 column (4.6 × 150 mm, 2.7 μM (Agilent Technology, Milan, Italy)), thermostated at 30 °C. Elution was carried out at a flow rate of 0.6 mL/min, using a mixture of 0.2% formic acid in methanol (A) and 0.2% formic acid in water (B) as the mobile phase at the following gradient: 0–6 min 10% A, 20 min 35% A, 46 min 40% A, 48 min 100% A, 60 min 100% A, 62 min 10% A, and 70 min 10% A. The injection volume was 20 µL. The HPLC system was coupled with a diode array detector (DAD) and an electrospray ionization mass detector (HPLC-DAD-ESI-MS^n^) in parallel by dividing the mobile phase 1:1. The acquisition was performed in full scan (*m*/*z* 50–1500) and full scan MS^2^ (*m*/*z* 50–600) by selecting the precursor ion [M-H]^−^ at *m/z* 289 for (+)-catechin and (−)-epicatechin, *m/z* 577 for procyanidin B2, *m/z* 353 for 5-*O*-caffeoylquinic acid (chlorogenic acid), *m/z* 163 for 3-hydroxycinnamic acid (IS), *m/z* 435– for phloridzin, *m/z* 463 for quercetin 3-*O*-galactoside, *m/z* 433 for quercetin xyloside and quercetin 3-*O*-arabinoside, *m/z* 447 for quercetin 3-*O*-rhamnoside, respectively. Analytes for which standards were not available, such as 3- and 4-*O*-caffeoylquinic acids ([M-H]^−^ at *m/z* 337); procyanidin B-type dimers ([M-H]^−^ at *m/z* 577), trimers ([M-H]^−^ at *m/z* 865), and tetramers ([M-H]^−^ at *m/z* 1153); caffeic acid hexoside ([M-H]^−^ at *m/z* 341); 4- and 5-*O*-p-Coumaroylquinic acids ([M-H]^−^ at *m/z* 337); phloretin-xyloglucoside ([M-H]^-^ at *m/z* 567); quercetin pentoside ([M-H]^−^ at *m/z* 433); and hexoside ([M-H]^−^ at *m/z* 463), were tentatively characterized by comparison of their fragmentation patterns (see Supplementary Materials, Table S1 and Figure S2) with those available in the literature.

Quantitative analysis of metabolites listed in Table 1 was performed using an Ultimate 3000 RS Diode Array detector (Thermo Fisher Scientific, MA, USA) controlled by Chromeleon software (version 6.80). Spectral data of all peaks were accumulated in the 200–600 nm range, and chromatograms were recorded at 280 nm for (+)-catechin, (−)-epicatechin, procyanidin B2, B-type procyanidins, phloretin derivatives, phloridzin, and 3-hydroxycinnamic acid (IS); at 328 nm for 3-, 4- and 5-*O*-caffeoylquinic acids, 4- and 5-*O*-*p*-coumaroylquinic acids, and caffeic acid hexoside; at 258 nm for quercetin 3-*O*-galactoside, quercetin-3-*O*-xyloside, quercetin-3-*O*-arabinoside, and quercetin glycosides; and at 520 nm for cyanidin derivative (see also Figure S1). Calibration curves (R^2^ > 0.999) were prepared by diluting a stock solution of each standard [(+)-catechin, (−)-epicatechin, procyanidin B2, 5-*O*-caffeoylquinic acid, phloridzin, quercetin 3-*O*-galactoside, quercetin-3-*O*-xyloside, and quercetin-3-*O*-arabinoside] in H_2_O/MeOH 9:1 (*v/v*) with 0.2% formic acid in the range of 12–3000 ng/mL with a constant concentration of IS (500 ng/mL). When standards were unavailable, the analyte was quantified using the calibration curve of the available standard with a similar chemical structure.

The procedure for the identification and quantification of β-sitosterol and triterpene compounds used in this study was described in detail in our previous works [10,11,12]. Briefly, the characterization of these secondary metabolites present in the Annurca apple extracts was carried out by GC–MS analysis of the corresponding trimethylsilyl derivatives (TMS). For this purpose, a Trace GC Ultra gas chromatograph equipped with a split/splitless injector and coupled to an ion-trap mass spectrometer detector Polaris Q (Thermo Scientific) was used. The column had the following specifications: 30 m × 0.25 mm i.d., 0.1 µM film thickness, and fused silica SLB-5ms (Supelco, Sigma-Aldich). The initial oven temperature was 240 °C, programmed to 280 °C at 2 °C/min and kept at 280 °C for 10 min; the temperature was then raised to 310 °C at a rate of 10 °C/min and maintained at this temperature for 20 min. Samples were injected in the split (1:10) mode. The injector, transfer line, and ion source were set at 280, 280, and 200 °C, respectively. Helium was used as carrier gas at a 1 mL/min flow. The mass spectra were recorded in electron ionization (EI) mode at 70 eV electron energy with a mass range from *m/z* 50 to 1000 and a scan rate of 0.8 scan/s.

The structural characterization of metabolites listed in Table 2 was carried out by comparison of the mass spectra of the chromatographic peaks with those of standards or to the spectra from the NIST02 spectral library, for β-sitosterol-TMS ([M]^+•^ at *m/z* 488), oleanolic acid-TMS ([M]^+•^ at *m/z* 600), ursolic acid-TMS ([M]^+•^ at *m/z* 600), uvaol-TMS ([M]^+•^ at *m/z* 586) or with those available in the literature for maslinic acid-TMS ([M]^+•^ at *m/z* 472), corosolic acid-TMS ([M]^+•^ at *m/z* 472), pomolic acid-TMS ([M]^+•^ at *m/z* 472), annurcoic acid-TMS ([M]^+•^ at *m/z* 486), and tormentic acid-TMS ([M]^+•^ at *m/z* 488).

The data acquisition was under the control of Xcalibur software (version 2.0.7; Thermo Scientific). The quantitative analysis was carried out using a Fisons GC 8000 series gas chromatograph equipped with a split/splitless injector and a flame ionization detector (Fisons Instruments, Milan, Italy). The separation was carried out with a fused silica capillary column DB-5MS UI 30 m × 0.250 mm × 0.25 μM film thickness (Agilent, J&W, Leini, Italy). The initial oven temperature was 240 °C programmed to 280 °C at 2 °C/min and kept at 280 °C for 25 min, the temperature was then raised to 310 °C at a rate of 10 °C/min and maintained at this temperature for 13 min. Samples (1 µL) were injected in the split (1:10) mode. The injector and detector were set at 280 °C. Hydrogen was used as carrier gas at a 1.8 mL/min flow. Peak areas were integrated using a Varian Galaxie Workstation (Agilent Technologies, Leini, Italy). Quantification of the triterpenic acids and β-sitosterol in the samples was performed using the internal standard method based on the relative peak area of analyte to IS (cholesterol) from the average of three replicate measurements. A calibration curve was built for each corresponding standard compound in the extracts. For this purpose, working solutions of each available standard (β-sitosterol, ursolic acid, and oleanolic acid) were prepared at a concentration ranging from 5 to 120 µg/mL with a constant concentration of the IS (cholesterol, 60.0 µg/mL). The calibration curves obtained had a high level of linearity with a correlation coefficient (*r*^2^) higher than 0.999 for all analytes. When standards were unavailable, the target analyte was quantified using the calibration curve of available standards of similar chemical structures.

### 2.5. DPPH Scavenging Activity

The assessment of antioxidant activity was conducted using the stable free radical DPPH• (2,2-diphenyl-1-picrylhydrazyl hydrate) following the methodology described by Saltarelli et al. [13]. In brief, 850 µL of a prepared 100 µM DPPH• ethanol solution was mixed with 150 µL of Annurca apple extracts at various concentrations (ranging from 0.46 mg/mL to 30 mg/mL) diluted in 80% EtOH. Subsequently, the reaction mixture was performed in darkness at room temperature for 30 min. A blank solution composed of 850 µL of DPPH• and 150 µL of 80% EtOH was employed as a reference. Notably, the analysis was performed on 96-well plates to facilitate multiple replicates per sample. The determination of the extracts’ DPPH radical scavenging ability (DSA) relied on the observation of reduced DPPH absorbance at 517 nm using the Bio-Rad microplate reader ‘Benchmark’ spectrophotometer.

DPPH Scavenging Activity (% DSA) = [A_517_ nm of blank − A_517_ nm of sample)/A_517_ nm of blank] × 100

Data are also reported as mg of ethanolic extract dry weight (dw) with 50% scavenging ability (EC50). 

### 2.6. ABTS Scavenging Activity

To measure the antioxidant activity against ABTS• (2,2′-azino-bis (3-ethylbenzothiazoline-6-sulphonic acid)) radical, the method described by Loizzo et al. [14] was followed with some modifications. First, the reaction mixture was prepared by combining 7 mM ABTS solution and 140 mM potassium persulphate and then incubated in the dark at room temperature for 12–16 h to create ABTS• radical. Before use, the solution was diluted with ethanol until an absorbance of 0.70 ± 0.02 at 734 nm was achieved. Then aliquots of Annurca apple extracts at concentrations ranging from 1.87 to 30 mg/mL were added to 1 mL of the ABTS ethanolic solution. The mixture was incubated in the dark at room temperature for 4 min. Subsequently, we measured the absorbance at 734 nm using a UV Beckman spectrophotometer (Palo Alto, CA, USA) and used ethanol as a blank. The ABTS radical scavenging activity was calculated following the equation:

ABTS Scavenging Activity (% ABTS SA) = [A_734_ nm of blank − A_734_ nm of sample)/A_734_ nm of blank] × 100.

Data are also reported as mg of ethanolic extract dry weight (dw) with 50% scavenging ability (EC50).

### 2.7. ORAC Assay

The antioxidant capacity of Annurca apple extracts was assessed using the ORAC (oxygen radical absorbance capacity) method, following previously published procedures [15], and employed a FLUOstar Optima Plate reader fluorimeter (BMG Labtech, Offenburg, Germany). The assay comprised 200 μL of 0.096 μM fluorescein in 0.075 M Na-phosphate buffer (pH 7.0), 20 μL of the sample or Trolox, or 0.075 M Na-phosphate buffer (pH 7.0) as the blank. The reaction was initiated with 40 μL of 0.33 M AAPH [2,2′azobis-(2-amidinopropane) hydrochloride]. Fluorescence intensity was measured at 485 nm (excitation) and 520 nm (emission) until complete extinction. A calibration curve using Trolox (ranging from 50 to 500 μM) in 0.075 M Na-phosphate buffer (pH 7.0) served as the positive standard. ORAC values, expressed as mmol Trolox equivalents (TEq)/100 mg dw, were obtained. Ten diluted callus extracts and twenty diluted pulp and peel extracts (30 mg/mL) were prepared for analysis.

### 2.8. Lipoxygenase Inhibition Assay

The assay involved the use of soybean 5-lipoxygenase following the protocol established by Saltarelli et al. [16]. Inhibition experiments were conducted to evaluate the impact on 5-lipoxygenase activity (0.18 μg/mL) using 100 μM linoleic acid as the substrate in a 50 mM sodium phosphate buffer at pH 6.8. The reaction mixture, in the absence of the enzyme, was pre-equilibrated at 20 °C for 20 min. Subsequently, inhibition studies were carried out in the presence of varying concentrations of Annurca apple extracts (ranging from 0.15 to 0.9 mg/mL). The measurements were recorded at 235 nm at 20 °C using a UV Beckman spectrophotometer. The percentage of lipoxygenase activity inhibition was calculated using the following formula:

% = 1 − {[(Δ_235_ nm of blank − Δ_235_ nm of sample)/Δ_235_ nm of blank] × 100}.

The determination of EC50 involved plotting a graph correlating the concentration of extracts with the percentage of inhibition of linoleic acid peroxidation.

### 2.9. Nicking Assay

The nicking assay was conducted following Sadat Asadi et al. [17] with slight adjustments. A mixture containing 125 ng/μL of pGEM-T DNA, freshly prepared H_2_O_2_ (5 mM), Fe_2_SO_4_ (0.33 mM), and EDTA (0.62 mM) was prepared. Annurca apple extracts at varying concentrations (ranging from 0.06 mg/mL to 4.5 mg/mL) were added to the mixture. The total volume was adjusted to 20 μL using phosphate-buffered saline (PBS = 8 g/L NaCl, 1.44 g/L Na_2_HPO_4_, 0.2 g/L KH_2_PO_4_, 0.2 g/L KCl, pH 7.4). A negative control containing DNA, EDTA, and PBS was also prepared. The reaction mixtures were then left to incubate for 30 min at 37 °C. Subsequently, the DNA samples were applied to a 1% agarose/tris borate EDTA (TBE) gel, stained using 1% Midori Green Advance DNA staining (Resnova, Genzano di Roma, Italy), and visualized under UV light using a Gel Doc 2000 (Bio-Rad, Italy). The quantification process involved densitometric analysis using Quantity One Software 4.01 (BioRad, Segrate, Italy). The retention of supercoiled DNA strands (%) was calculated using the following equation:

Retention % = (Intensity of supercoiled DNA of sample/Intensity of supercoiled DNA of control) × 100.

### 2.10. Cell Cultures

Murine RAW 264.7 macrophages were procured from the European Collection of Cell Cultures based in Salisbury, UK. These cells were cultured in Dulbecco’s Modified Eagle’s Medium (DMEM, Sigma-Aldrich, Milan, Italy), supplemented with 10% (*v/v*) heat-inactivated fetal bovine serum, 100 mM non-essential amino acids, streptomycin (100 μg/mL), penicillin (100 U/mL), and 1% (*w/v*) L-glutamine. The cells were maintained in a humidified incubator with 95% humidity at 37 °C with 5% CO_2_. The growth medium was replenished every 2–3 days until approximately 80% confluence was achieved.

### 2.11. Griess Assay

The production of nitric oxide (NO) by RAW 264.7 cells following stimulation with bacterial lipopolysaccharide (LPS, Sigma-Aldrich, Milan, Italy) serves as an established indicator of the inflammatory process. The extracellular NO concentration was determined using a UV–visible spectrophotometer. The nitric oxide content was quantified by referencing a nitrite standard curve (calcium nitrite 6.25–100 μM) that had been prepared in the fresh culture medium.

RAW 264.7 cells were seeded in the logarithmic phase of 3 × 10^4^ cells/100 μL and cultured in 96-well plates for 24 h. The experiment was then divided into four groups: control group (CTRL), lipopolysaccharide (LPS)-treated group, LPS + dexamethasone (DEXA, an anti-inflammatory compound used as a positive control), and LPS + extracts at various concentrations. The treatments were performed for 24 h at 37 °C. LPS was used at a concentration of 1 μg/mL and DEXA at 0.0039 mg/mL. Following overnight treatments, 50 μL aliquots of supernatants were combined with 50 μL of Griess reagent (40 mg/mL) and incubated for 10 min in the absence of light at room temperature (RT). The absorbance was measured at 570 nm employing a plate reader (BioRad Laboratories, Hercules, CA, USA).

To eliminate the potential association between the decrease in NO levels and a reduction in cell viability, we performed the MTT–viability assay on the same samples.

### 2.12. Cell Viability Assay

Cell viability was assessed using 3-(4,5-dimethylthiazol-2yl)-2,5-diphenyl tetrazolium bromide (MTT) assays. Following removal of the medium, cells were washed with PBS and each well was treated with a 100 µL di MTT 0.2 mg/mL solution for 1 h at 37 °C. Following the incubation phase, the MTT solution was extracted, and 100 µL of dimethyl sulfoxide (DMSO) was introduced to facilitate the dissolution of the resultant formazan salt. The quantification of formazan salt was carried out by gauging the absorbance at 570 nm and employing a microplate reader. Cell viability was expressed as a percentage compared to the control and calculated using the formula:

% Cell viability = (OD test/OD control) × 100.

where OD represents optical density.

### 2.13. Statistical Analyses

The statistical analyses were performed using GraphPad Software (GraphPad Prism version 6 for Windows). Values were expressed either as the mean ± standard error of the mean (SEM) or standard deviation (SD). The treated and control sample variables were compared using one-way ANOVA. Dunnett’s multiple comparisons test was employed to calculate the significant difference. The differences between samples were considered significant if *p* values were <0.05.

## 3. Results and Discussion

### 3.1. Callus Induction from Annurca Fruit Pulp and Chemical Characterization of Annurca Ethanolic Extracts

**Figure 1:**
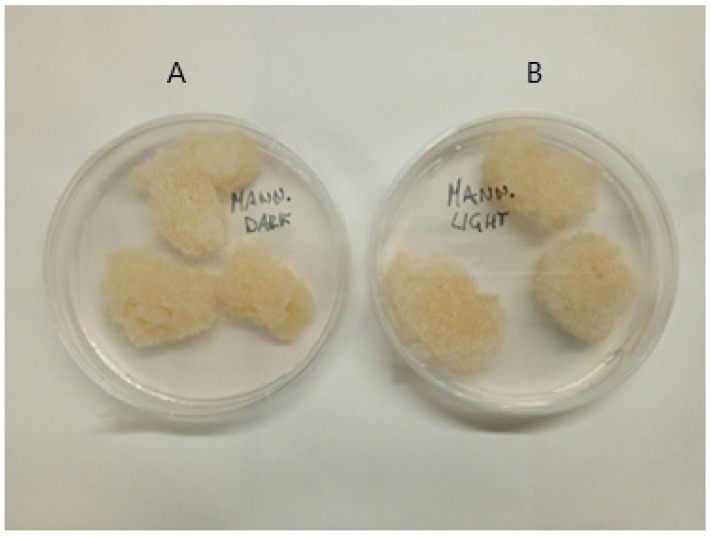
Annurca apple pulp-derived calli. (**A**) Callus grown in the dark (‘dark callus’); (**B**) callus maintained in an 18-h photoperiod (‘light callus’).

The literature on the properties of the Annurca apple is extensive [18,19,20,21], but there is limited knowledge about callus cultures from any organ of this apple variety [22]. Based on our previous experience with various callus cultures obtained from Mela Rosa Marchigiana, Golden Delicious [10], Red Sentinel (patent n. 102020000012466), *Acca Sellowiana* Burret exotic fruit [11], and *Cydonia oblonga* Mill [12], in the present work, two callus cultures (DC and LC) derived from the pulp of Annurca apples were obtained as reported in the Section 2. The experimental conditions developed in this work allowed, after two weeks, for the formation of friable, cream-colored calli above and around the explant on the cut surface, both in the culture kept in the dark and under an 18-h photoperiod. In both conditions, to allow for regular and lasting callus production, it was also necessary to add cytokinin (BA) to the culture medium, following what has been reported in previously published works [10,11,12,22]. The formation of calli in the two distinct culture conditions was documented after two months of cultivation as in Verardo et al. [10], considering the fresh and dry weight of callus obtained in each experimental condition. It is known that the development of callus culture and the production of bioactive secondary metabolites are influenced by the frequency and timing of light exposure [23,24,25,26,27,28,29]: however, no quantitatively significant differences were found in the weight of callus produced under the two conditions used in this work. Nor were any differences found in the texture, color, and shape of the callus obtained in the two culture conditions (Figure 1).

Moreover, the production of secondary metabolites is comparable. The characterization and the quantification of phenolic compounds were performed by HPLC-DAD-ESI-MS^n^. As reported in Table 1, the highest total phenolic content was found in peel, followed by pulp and LC, whereas DC showed the lowest values (111 times lower with respect to peel). In the peel, the most represented phenolic compound was quercetin 3-O-galactoside (66.40 µg/100 mg dw ~16% of its total phenolic compounds), followed by chlorogenic acid (49.32 µg/100 mg dw ~12% of its total phenolic compounds) and (−) epicatechin (44.62 µg/100 mg dw ~11% of its total phenolic compounds). The pulp, on the other hand, had a considerable and higher chlorogenic acid content (61.97 µg/100 mg dw ~40% of its total phenolic compounds) than the peel, attended by (−) epicatechin (25.09 µg/100 mg dw ~16% of its total phenolic compounds) and other several compounds presented at low concentrations. Procyanidin B2 was found in both peel and pulp, with the highest concentrations in the peel but, was absent in both calli. Chlorogenic acid was the most represented compound in the LC (19.57 µg/100 mg dw ~69% of its total phenolic compounds).

GC-MS analysis enabled the identification of β-sitosterol and triterpene compounds that were mainly present in the calli, less in the peel, and negligible in the pulp (Table 2). Tormentic acid (>45%) was predominantly produced in both calli in very similar concentrations, followed by corosolic (13% and 9% in LC and DC, respectively), maslinic (~12%), annurcoic (10% and 17% in LC and DC, respectively), and ursolic acid (>7%). In the peel, ursolic acid prevails with a percentage of about 67.06%, along with oleanoic (~13%) and annurcoic acids (~9%).

The inclusion of plant growth regulators (PGRs) within culture media has been identified as a significant factor influencing the production of secondary metabolites (SMs), as evidenced in the existing literature. For instance, triterpenes like betulinic acid, ursolic acid, and oleanolic acid, which are extracted from callus cultures of different sage species, can have variations based on the plant growth regulators (PGRs) used and the specific plant parts (such as stems or leaves) used to initiate the callus production. It has been demonstrated that callus obtained from leaves or stems of plants generally produces a lower concentration of secondary metabolites compared to the original plant. This difference in metabolite production could be attributed to the organization and cellular differentiation [30]. It is possible that mature apple pulp cells in culture, like in our case, may have different gene expression regulation compared to the peel and pulp of the original fruit, leading to a substantial increase in the biosynthesis and accumulation of bioactive compounds, particularly triterpene compounds, as in this instance.

In summary, both calli produced high levels of triterpene acids (mainly tormentic, corosolic, maslinic, and ursolic acids) and low levels of polyphenols, which are more abundant in peel and pulp extracts (Table 1 and Table 2). Triterpenic acids in nature are primarily found on plant surfaces such as fruit peels, stem bark, leaves, and fruit waxes [31], but they are very poorly identified in the fruit pulp. No triterpene acids were found by Laezza’s group [22], which showed that yeast extract added to the culture medium to produce callus from Annurca campana pulp explants induced a significant increase in total polyphenol content.

The most abundant triterpene in both callus cultures studied in this work was tormentic acid. This compound is a pentacyclic triterpene that can regulate insulin production, has a hypoglycemic effect, and exhibits antioxidant and anti-inflammatory properties that are beneficial in healing cardiovascular disorders [32,33]. Following Babich et al. [34], alterations to the nutrient medium have the potential to bolster the production of biologically active substances by stimulating their biosynthesis. Considering the established bioeffect of this triterpene, our objective is to identify the specific medium nutrients capable of enhancing biosynthesis and yield. This initiative will facilitate the utilization of callus cultures as a reliable source of tormentic acid.

### 3.2. Antioxidant Capacity of Annurca Apple Extracts

**Figure 2:**
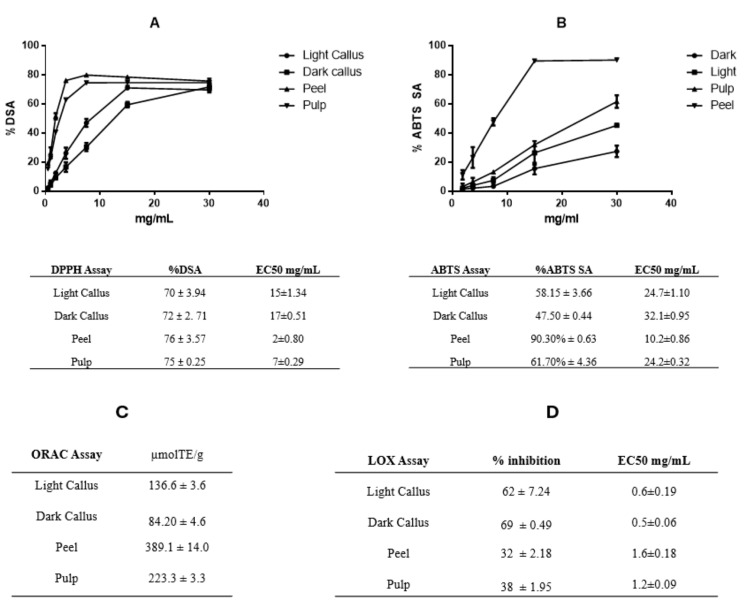
DPPH (**A**), ABTS (**B**), ORAC (**C**) tests, and lipoxygenase inhibition activity (**D**). The data represent the percentage of inhibition induced by increasing concentrations of Annurca apple extracts. Each value is the mean ± SD of three independent measurements.

The different Annurca apple extracts were tested for their antioxidant activity with the use of different assays. DPPH, ABTS^+^, and ORAC assays were used to investigate free radical scavenging activity. As indicated in Figure 2A, all extracts exhibited a radical scavenging activity against hydroxyl radicals (DPPH scavenging activity—%DSA) in a dose-dependent manner with inhibition ranging from 70 to 76% in all four extracts at a concentration of 30 mg/mL. At lower concentrations, peel and pulp extracts were greater in DPPH scavenging activity compared to callus extracts, demonstrated by their lowest EC50 value (2 ± 0.80 and 7± 0.29 mg/mL in peel and pulp, respectively) (Figure 2A).

The maximum ABTS scavenging activity (ABTS SA) recorded at a concentration of 30 mg/mL was 58.15% ± 3.66, 47.50% ± 0.44, 90.30% ± 0.63, 61.70% ± 4.36 for LC, DC, peel, and pulp, respectively (Figure 2B).

Finally, the ORAC assay confirmed the good antioxidant activity of different Annurca apple extracts showing values ranging from 84.20 ± 4.6 to 136.6 ± 3.6 for DC and LC, and 223.3 ± 3.3 to 389.1 ± 14.0 μM Trolox equivalents/100mg for pulp and peel, respectively (Figure 2C).

The higher scavenging activity demonstrated by DPPH, ABTS, and ORAC assays from *in vivo*-derived plant extracts (peel and pulp extracts) may be due to the extracts’ higher total amount of phenolic compounds compared to callus extracts. A linear relationship between the antioxidant effect and the amount of phenolic compounds has been demonstrated [35]. In fact, the peel extract has the highest amount of polyphenols, which corresponds to the highest scavenging activity compared with the other extracts.

We also looked at how Annurca callus (DC and LC), peel, and pulp extracts inhibited lipoxygenase-catalyzed linoleic acid oxidation. We examined concentrations ranging from 0.15 to 0.9 mg/mL and found that all extracts inhibit the oxidation of linoleic acid in a dose-dependent way. At the highest concentration of 0.9 mg/mL, DC has the greatest inhibiting activity (69%), followed by LC (62%), the pulp (38%), and the peel (32%) (Figure 2D).

The presence of triterpenes in the callus extracts can explain why they inhibit the oxidation of linoleic acid more effectively than peel and pulp [36,37,38,39]. 5-lipoxygenase (5-LO) is an enzyme that converts free arachidonic acid into leukotrienes (LTs), which have been linked to a variety of medical conditions including asthma, allergic rhinitis, inflammatory bowel and dermatological pathologies, rheumatoid arthritis, cancer, osteoporosis, and cardiovascular disease. As a result, pharmaceutical therapies that disrupt the 5-LO pathway demonstrate potential therapeutic efficacy for many related illnesses [40]. Therefore, callus cultures of Annurca apple pulp, being rich in triterpene acids, can be considered an important source of substances with anti-inflammatory activity.

### 3.3. Genoprotective Activity of Annurca Apple Extracts

**Figure 3:**
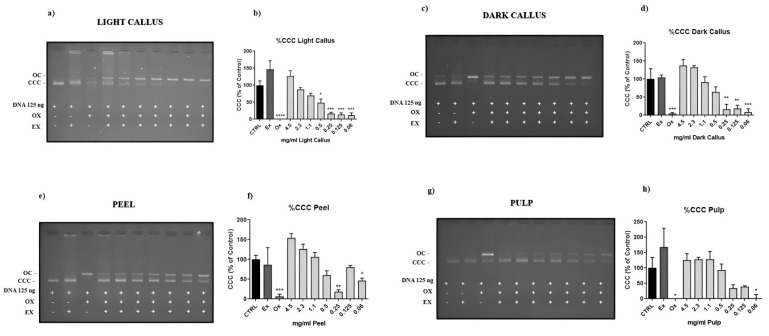
Protective effect of Annurca apple extracts against strand breaks using the DNA nicking assay. In (**a**,**c**,**e**,**g**), an example of agarose gel of the pGEM plasmid after incubation with the oxidant system in the presence of decreasing amounts of extract; in (**b**,**d**,**f**,**h**), the quantification of gels obtained by the assay, expressed as the percentage ratio between the volume of the band of the plasmid supercoiled form (CCC) after incubation with oxidant and the same band volume in the control; each value represents the mean ± SEM of three independent measurements. (* *p* < 0.05; ** *p* < 0.01; *** *p* < 0.001; **** *p* < 0.0001; Dunnett’s post hoc test).

The DNA nicking assay was performed to assess the DNA-protective efficacy of various Annurca apple extract concentrations. On agarose gel electrophoresis, the plasmid DNA is separated into three bands. The native supercoiled circular DNA (CCC) is the quickest-moving band, the center band corresponds to linear DNA (L DNA), and the slowest-moving band is the open circular form (OC), in which DNA is broken on one strand. Compared to the plasmid DNA control, the CCC form is changed to the OC form by the hydroxyl radical (•OH) that caused DNA damage. Annurca apple extracts are able to reduce this damage. Nicking assays demonstrated that DC could protect DNA better than LC, but pulp has the greatest protection (Figure 3).

The results obtained from this assay are important because DNA damage is linked to several illnesses, including cancer, neurological disorders, and aging [41,42,43]. However, further research will be undertaken to demonstrate the genoprotective efficacy of Annurca apple extracts in cellular models.

### 3.4. Anti-Inflammatory Activity of Annurca Apple Extracts

**Figure 4:**
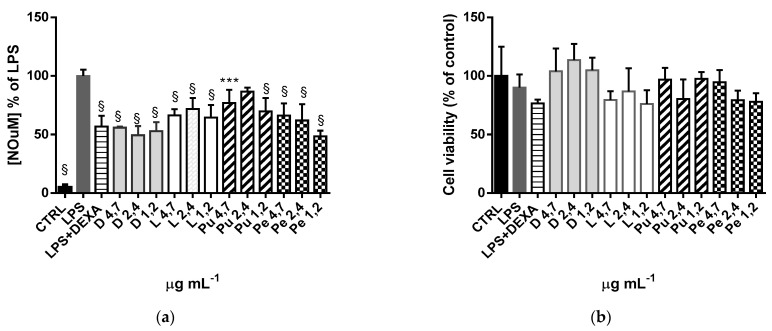
Evaluation of extracellular NO release and cell viability of RAW 264.7 cells after LPS, Annurca extracts, and DEXA treatment. (**a**) RAW 264.7 cells were co-treated overnight with LPS 1 µg mL^−1^ either in the absence or presence of Annurca extracts at different concentrations or DEXA 0.0039 mg mL^−1^. CTRL: untreated cells. The results are expressed as NO reduction compared to LPS-treated cells (*** *p* < 0.001, § *p* < 0.0001); ANOVA followed by Dunnett’s multiple comparison test was performed). (**b**) Cell viability assay on Raw 264.7 cells co-treated with LPS and Annurca extracts (MTT). The results are expressed as % of CTRL ± SD of three independent. ANOVA followed by Dunnett’s multiple comparison test was performed; No statistical differences were found.

Since the experiments described above showed that DC and LC extracts inhibited lipoxygenase-catalyzed linoleic acid oxidation more than pulp and peel extracts, suggesting a possible anti-inflammatory activity by these extracts (Figure 2D), we decided to explore the anti-inflammatory activity of Annurca apple extracts. RAW264.7 cells were chosen as a suitable macrophage model with which it is possible to quickly evaluate inflammatory cell responses with the Griess test [44], which evaluates NO release after treatment with bacterial LPS. The anti-inflammatory molecule DEXA was used as a positive control. The range of concentration was previously selected by MTT cell viability assay. From the results in Figure 4a, the overnight co-treatment with 1 µg/mL of LPS and extracts at different concentrations significantly reduces NO release. In contrast to the evidence shown in the lipoxygenase inhibition assay, all the extracts showed anti-inflammatory activity with the same statistical significance value. To rule out the possibility that NO reduction resulted from a decrease in RAW 264.7 cell viability following LPS treatment, a cell viability assay (MTT) was performed. Figure 4b shows that co-treated (LPS with DEXA or extract) cells are comparable to untreated ones (CTRL).

Thus, our results demonstrate that all the extracts have no cytotoxic effect on RAW 264.7 cells and possess a potent anti-inflammatory property. The lack of statistical differences between the extracts could be attributed to the fact that both triterpenes and polyphenols have anti-inflammatory properties [45,46,47,48,49,50], and they could exert a synergistic effect in all four extracts. Further experiments will be conducted to investigate if reducing the concentration of the Annurca apple extracts results in a statistically significant difference in their activity.

## 4. Conclusions

The results presented in this work show that the callus culture technique allows for the production of high quantities of SMs starting from a small portion of the desired plant organ keeping the environmental parameters under control and preserving the biodiversity of the various plant species. Different combinations of media and plant growth regulators, as well as the modulation of other parameters (for example, light), could lead to the accumulation of specific SMs. In this work, the parameters selected to produce the two calli allowed us to accumulate large quantities of triterpenic compounds never detected in apple pulp. Preliminary results presented in this work regarding the biological activity of callus-derived extracts show that both calli can exert mainly anti-inflammatory and genoprotective effects and lower antioxidant activity. These results encourage the use of these extracts as nutraceutical or cosmeceutical-enriched products.

It will also encourage the development of different *in vitro* callus culture protocols from the same starting plant material, in this case apple pulp, to utilize them as bioreactors of classes or specific secondary metabolites that could be used as functional food for human well-being.

## Figures and Tables

**Table 1:** Identification and quantification (µg/100 mg dw ± SD) of phenolic compounds in the extracts of light callus, dark callus, peel, and pulp of Annurca apple by HPLC-DAD-ESI-MS^n^.

Compound	Light Callus	Dark Callus	Peel	Pulp
3-*O*-Caffeoylquinic acid isomer	0.05 ± 0.001	n.d.	0.18 ± 0.001	0.12 ± 0.001
Procyanidin B-type dimer isomer	n.d.	n.d.	7.38 ± 0.011	6.77 ± 0.006
Procyanidin B-type trimer isomer	n.d.	n.d.	3.87 ± 0.007	1.69 ± 0.008
Procyanidin B-type trimer isomer	n.d.	n.d.	1.67 ± 0.018	0.90 ± 0.022
Procyanidin B-type tetramer isomer	n.d.	n.d.	0.83 ± 0.012	0.42 ± 0.001
Procyanidin B-type trimer isomer	n.d.	n.d.	4.27 ± 0.018	1.93 ± 0.021
4-*O*-Caffeoylquinic acid	0.01 ± 0.001	n.d.	0.29 ± 0.005	0.09 ± 0.001
(+) Catechin	n.d.	n.d.	8.37 ± 0.035	7.32 ± 0.019
Procyanidin B-type tetramer isomer	n.d.	n.d.	3.93 ± 0.008	1.24 ± 0.009
Procyanidin B2	n.d.	n.d.	23.26 ± 0.152	10.18 ± 0.021
5-*O*-Caffeoylquinic acid (chlorogenic acid)	19.57 ± 0.034	0.10 ± 0.001	49.32 ± 0.110	61.97 ± 0.061
Caffeoyl acid hexoside	2.16 ± 0.005	3.46 ± 0.009	0.56 ± 0.003	0.39 ± 0.004
4-*O*-Caffeoylquinic acid isomer	0.43 ± 0.001	n.d.	1.02 ± 0.001	0.84 ± 0.001
Procyanidin B-type trimer isomer	n.d.	n.d.	15.32 ± 0.098	6.93 ± 0.011
4-*O*-*p*-Coumaroylquinic acid isomer	0.32 ± 0.003	n.d.	0.98 ± 0.011	0.46 ± 0.002
Cyanidin hexoside	n.d.	n.d.	12.27 ± 0.181	n.d.
(-) Epicatechin	0.17 ± 0.002	0.07 ± 0.001	44.62 ± 0.324	25.09 ± 0.093
5-*O*-Caffeoylquinic acid isomer	0.51 ± 0.001	n.d.	1.53 ± 0.001	1.79 ± 0.005
5-*O*-*p*-Coumaroylquinic acid isomer	0.29 ± 0.002	0.01 ± 0.001	1.08 ± 0.001	1.24 ± 0.001
4-*O*-*p*-Coumaroylquinic acid isomer	0.44 ± 0.004	n.d.	2.84 ± 0.006	5.55 ± 0.013
5-*O*-*p*-Coumaroylquinic acid isomer	0.04 ± 0.001	n.d.	0.45 ± 0.007	0.15 ± 0.002
Procyanidin B-type dimer isomer	n.d.	n.d.	2.41 ± 0.002	1.02 ± 0.003
Quercetin 3-*O*-galactoside	1.93 ± 0.020	n.d.	66.40 ± 0.151	0.14 ± 0.001
Phloretin xyloglucoside isomer	n.d.	n.d.	23.84 ± 0.034	15.26 ± 0.016
Quercetin hexoside	1.41 ± 0.001	n.d.	25.80 ± 0.037	0.79 ± 0.001
Quercetin 3-*O*-xyloside	0.25 ± 0.001	n.d.	14.60 ± 0.112	0.36 ± 0.004
Phloretin xyloglucoside isomer	n.d.	n.d.	1.12 ± 0.010	0.60 ± 0.005
Quercetin 3-*O*-arabinoside	0.03 ± 0.001	n.d.	6.60 ± 0.075	0.03 ± 0.001
Phloridzin	0.63 ± 0.010	n.d.	36.21 ± 0.061	1.71 ± 0.005
Quercetin pentoside	0.10 ± 0.001	n.d.	28.86 ± 0.064	0.28 ± 0.001
Quercetin 3-*O*-rhamnoside	0.04 ± 0.001	n.d.	13.23 ± 0.143	0.91 ± 0.009
Total	28.39 ± 0.003	3.64 ± 0.011	403.12 ± 0.493	156.16 ± 0.094

Data are expressed as the mean value; n = 2 repetitions. n.d.: not detected. The presence of the same isomers repeated several times in the table shows that the molecules had the same fragmentation pattern but different retention times.

**Table 2:** Identification and quantification (µg/100 mg dw ± SD) of β-sitosterol and triterpene compounds in the extracts of light callus, dark callus, peel, and pulp of Annurca apple, by GC-MS and GC-FID.

Compound	Light Callus	Dark Callus	Peel	Pulp
β-Sitosterol	148.52 ± 0.85	174.69 ± 1.47	44.61 ± 0.46	32.04 ± 0.25
Uvaol	13.45 ± 0.04	13.13 ± 0.13	n.d.	n.d.
Oleanolic acid	49.80 ± 0.45	34.92 ± 0.18	163.91 ± 0.83	3.95 ± 0.02
Ursolic acid	210.40 ± 1.29	161.48 ± 0.43	864.08 ± 0.90	21.40 ± 0.19
Maslinic acid	276.61 ± 0.88	269.74 ± 2.08	17.78 ± 0.13	n.d.
Corosolic acid	321.53 ± 0.84	201.67 ± 0.88	42.71 ± 0.22	n.d.
Pomolic acid	14.88 ± 0.01	16.57 ± 0.06	50.46 ± 0.27	n.d.
Annurcoic acid	251.89 ± 2.29	381.55 ± 2.54	128.36 ± 0.56	1.31 ± 0.01
Tormentic acid	1152.75 ± 10.35	1032.97 ± 7.05	16.25 ± 0.23	0.42 ± 0.01
Σ triterpenic acid	2277.85 ± 7.16	2098.91 ± 7.30	1283.55 ± 0.87	27.08 ± 0.22
Total	2439.82 ± 8.05	2286.73 ± 8.64	1328.15 ± 1.32	59.12 ± 0.47

Data are expressed as the mean value; n = 2 repetitions. n.d.: not detected.

## Data Availability

The original contributions presented in the study are included in the article/supplementary material, further inquiries can be directed to the corresponding author.

## Supplementary Materials

The following supporting information can be downloaded at: https://www.mdpi.com/article/10.3390/foods13132036/s1, Figure S1. Representative HPLC-DAD chromatograms of purified sample of Annurca apple peel ethanolic extract detected at 280, 328, 258 and 520 nm. Figure S2. Representative HPLC-ESI-MS^n^ extracted ion chromatograms (EIC) of the most abundant phenolic compounds identified in the sample of Annurca apple peel ethanolic extract. On the right of the figure are reported, for each analyte, the precursor ion [M-H]^−^. Table S1. Characterization of the main phenolic compounds found in the purified samples of Annurca apple ethanolic extracts by HPLC-DAD/ESI-MS^n^ in negative mode.
