# Supplementary material for: Anti-Inflammatory, Antioxidant, and Genoprotective Effects of Callus Cultures Obtained from the Pulp of Malus pumila cv Miller (Annurca Campana Apple)
Source: Foods. 2024 Jun 27;13(13):2036. doi: 10.3390/foods13132036 (PMC11241768; doi:10.3390/foods13132036)
Supplement: Supplementary file 1 [file foods-13-02036-s001.zip › foods-3078323-supplementary.pdf]

Anti-inflammatory, anti-oxidant, and genoprotective effects of Callus cultures from the pulp of Malus pumila cv Miller (Annurca Campana apple)

Supplementary material

Federica Gubitosa[a], Daniele Fraternale[a], Leila Benayada[a], Roberta De Bellis[a], Andrea Gorassini[b], Roberta Saltarelli[a], Sabrina Donati Zeppa[a], Lucia Potenza*.

[a] Department of Biomolecular Sciences, University of Urbino Carlo Bo, 61029 Urbino, Italy

[b] Department of Humanities and Cultural Heritage, University of Udine, 33100 Udine, Italy

Corresponding Author

Lucia Potenza –

Associate Professor of Biochemistry

University of Urbino Carlo Bo

Department of Biomolecular Sciences

School of Pharmacy

Campus Scientifico Sogesta

Via Ca' Le Suore, 2- 61029 Urbino (PU)

Tel : +39 (0)722-303803

Fax :+39 (0)722-305324

Email: lucia.potenza@campus.uniurb.it

Analysis of extracts from peel, pulp, and calli of Annurca apple by HPLC-DAD-ESI-MS^n

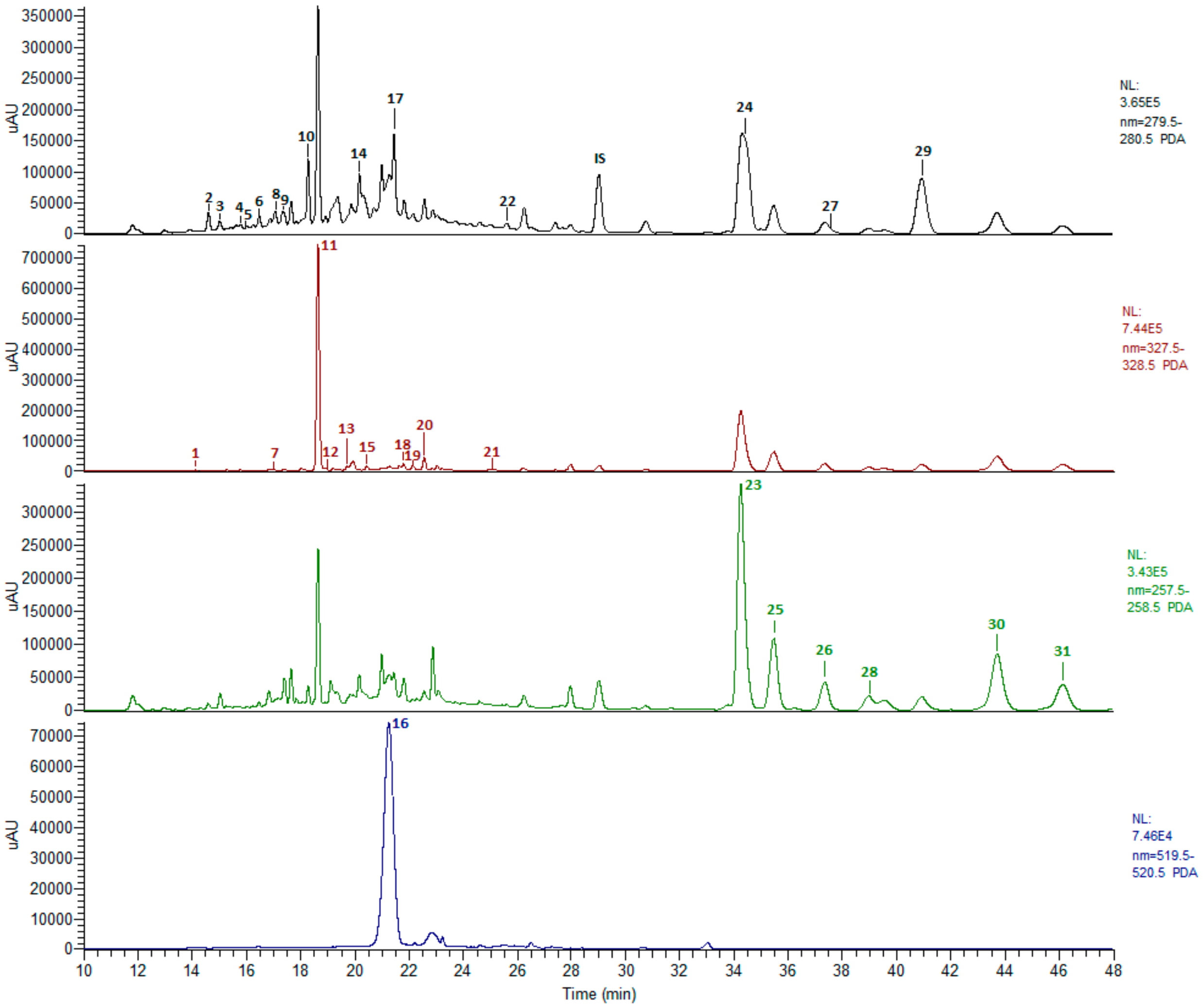

Figure S1. Representative HPLC-DAD chromatograms of purified sample of Annurca apple peel ethanolic extract detected at 280, 328, 258 and 520 nm. Peak numbers correspond to those reported in Tables S1.

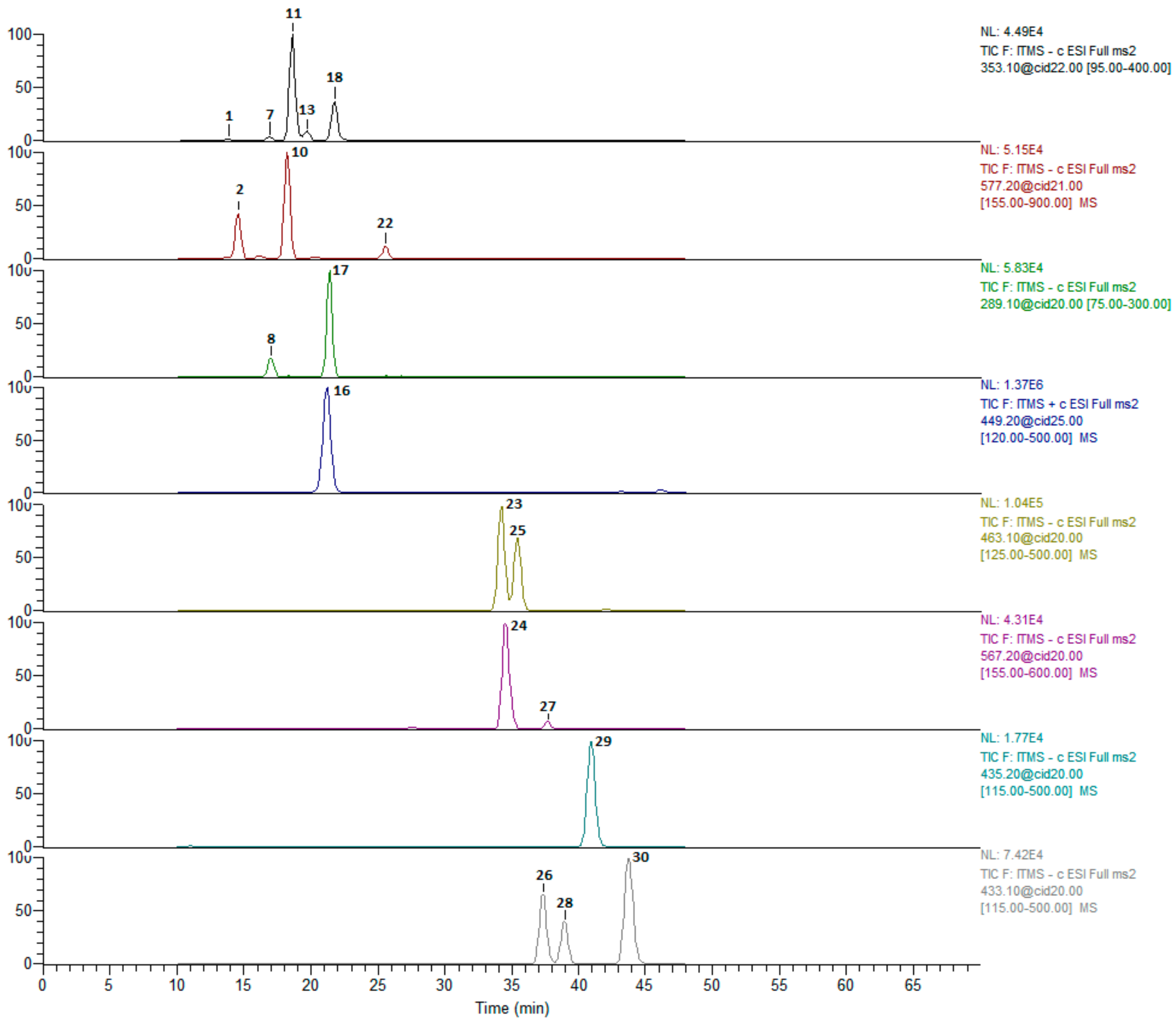

Figure S2.
Representative HPLC-ESI-MSn extracted ion chromatograms (EIC) of the most abundant phenolic compounds identified in the sample of Annurca apple peel ethanolic extract. On the right of the figure are reported, for each analyte, the precursor ion $[M- H]^-$. Peak numbers correspond to those reported in Tables S1.

Table S1. Characterization of the main phenolic compounds found in the purified samples of Annurca apple ethanolic extracts by HPLC-DAD/ESI-MS^n in negative mode.

Peak No	t_R (min)	Tentative assignment	Molar mass (g/mol)	Molecular Formula	λ_{max} (nm)	$[M-H]^-$ (m/z)	HPLC-ESI/MS^n *m/z* (% base peak)
1	13.9	3-*O*-Caffeoylquinic acid isomer	354.3	$C_{16}H_{18}O_9$	328	353	MS^2[353]: 191 (100), 179 (44), 135 (8)
2	14.6	Procyanidin B-type dimer isomer	578.5	$C_{30}H_{26}O_{12}$	280	577	MS^2[577]: 425 (100), 451 (30), 407 (24), 289 (18)
3	15.0	Procyanidin B-type trimer isomer	866.8	$C_{45}H_{38}O_{18}$	280	865	MS^2[865]: 739 (100), 577 (65), 847 (70), 695 (50), 425 (20), 407 (12)
4	15.8	Procyanidin B-type trimer isomer	866.8	$C_{45}H_{38}O_{18}$	280	865	MS^2[865]: 695 (100), 739 (90), 847 (80), 577 (40), 407 (20), 425 (10)
5	16.0	Procyanidin B-type tetramer isomer	1155.0	$C_{60}H_{50}O_{24}$	280	1153	MS^2[1153]: 863(100), 1089 (85), 1009 (71), 865(41), 847(38), 577 (41), 1027 (28), 425 (20), 1135 (11)
6	16.5	Procyanidin B-type trimer isomer	866.8	$C_{45}H_{38}O_{18}$	280	865	MS^2[865]: 739 (100), 695 (95), 847 (60), 577 (48), 407 (20), 425 (18)
7	16.9	4-*O*-Caffeoylquinic acid isomer	354.3	$C_{16}H_{18}O_9$	328	353	MS^2[353]: 173 (100), 179 (50), 191 (13), 135 (5)
8	17.1	(+) Catechin[a]	290.3	$C_{15}H_{14}O_6$	280	289	MS^2[289]: 245 (100), 205 (26), 179 (14), 231 (6), 203(5)
9	17.3	Procyanidin B-type tetramer isomer	1155.0	$C_{60}H_{50}O_{24}$	280	1153	MS^2[1153]: 865 (100), 863 (80), 1089 (73), 1009 (65), 1027 (50), 577 (45), 847 (35), 1135 (18), 425 (14)
10	18.3	Procyanidin B2[a]	578.5	$C_{30}H_{26}O_{12}$	280	577	MS^2[577]: 425 (100), 407 (24), 451 (18), 289 (10)
11	18.6	5-*O*-Caffeoylquinic acid[a]	354.3	$C_{16}H_{18}O_9$	328	353	MS^2[353]: 191 (100), 179 (8), 135 (1)
12	18.9	Caffeic acid hexoside	342.3	$C_{15}H_{18}O_9$	328	341	MS^2[341]: 179 (100), 135 (5)
13	19.7	4-*O*-Caffeoylquinic acid isomer	354.3	$C_{16}H_{18}O_9$	328	353	MS^2[353]: 173 (100), 179 (56), 191 (20), 135 (5)
14	20.2	Procyanidin B-type trimer isomer	866.8	$C_{45}H_{38}O_{18}$	280	865	MS^2[865]: 739 (100), 695 (90), 847 (62), 577 (52), 425 (16), 407 (14)
15	20.4	4-O-*p*-Cumaroylquinic acid isomer	338.3	$C_{16}H_{18}O_8$	328	337	MS^2[337]: 173 (100), 163 (10)
16	21.3	Cyanidin hexoside [b]	449.4	$C_{21}H_{21}O_{11}^+$	520	449^+	MS^2[449]: 287 (100)
17	21.4	(-) Epicatechin[a]	290.3	$C_{15}H_{14}O_6$	280	289	MS^2[289]: 245 (100), 205 (28), 179 (15), 231 (8), 203(6)
18	21.8	5-*O*-Caffeoylquinic acid isomer	354.3	$C_{16}H_{18}O_9$	328	353	MS^2[353]: 191 (100), 179 (9), 135 (1)
19	22.1	5-O-*p*-Cumaroylquinic acid isomer	338.3	$C_{16}H_{18}O_8$	328	337	MS^2[337]: 191 (100), 163 (5)
20	22.6	4-O-*p*-Cumaroylquinic acid isomer	338.3	$C_{16}H_{18}O_8$	328	337	MS^2[337]: 173 (100), 163 (10)
21	25.0	5-O-*p*-Cumaroylquinic acid isomer	338.3	$C_{16}H_{18}O_8$	328	337	MS^2[337]: 191 (100), 163 (5)
22	25.6	Procyanidin B-type dimer isomer	578.5	$C_{30}H_{26}O_{12}$	280	577	MS^2[577]: 425 (100), 451 (26), 407 (22), 289 (15)
23	34.3	Quercetin 3-O-galactoside[a]	464.4	$C_{21}H_{20}O_{12}$	258	463	MS^2[463]: 301 (100)
24	34.5	Phloretin xyloglucoside isomer	568.5	$C_{26}H_{32}O_{14}$	280	567	MS^2[567]: 273 (100)
25	35.5	Quercetin hexoside	464.4	$C_{21}H_{20}O_{12}$	258	463	MS^2[463]: 301 (100)
26	37.4	Quercetin 3-*O*-xyloside[a]	434.3	$C_{20}H_{18}O_{11}$	258	433	MS^2[433]: 301 (100)
27	37.7	Phloretin xyloglucoside isomer	568.5	$C_{26}H_{32}O_{14}$	280	567	MS^2[567]: 273 (100)
28	38.9	Quercetin 3-*O*-arabinoside[a]	434.3	$C_{20}H_{18}O_{11}$	258	433	MS^2[433]: 301 (100)
29	41.0	Phloridzin[a]	436.4	$C_{21}H_{24}O_{10}$	280	435	MS^2[435]: 273 (100)
30	43.8	Quercetin pentoside	434.3	$C_{20}H_{18}O_{11}$	258	433	MS^2[433]: 301 (100)
31	46.2	Quercetin 3-*O*-rhamnoside[a]	448.4	$C_{21}H_{20}O_{11}$	258	447	MS^2[447]: 301 (100), 300 (20), 285 (10)

[a]Confirmed with standard. [b]Detected in positive mode as M^+.
